# Population level mitogenomics of long-lived bats reveals dynamic heteroplasmy and challenges the Free Radical Theory of Ageing

**DOI:** 10.1038/s41598-018-31093-2

**Published:** 2018-09-11

**Authors:** David Jebb, Nicole M. Foley, Conor V. Whelan, Frédéric Touzalin, Sebastien J. Puechmaille, Emma C. Teeling

**Affiliations:** 1 School of Biology and Environmental Science, University College Dublin, Belfield, Dublin 4, Ireland; 2 Applied Zoology and Conservation, Zoological Institute, Greifswald University, Greifswald, Germany; 3 Laboratoire Evolution et Diversité Biologique, Université Toulouse 3, Paul Sabatier, 31062 Toulouse Cedex 09 France

## Abstract

Bats are the only mammals capable of true, powered flight, which drives an extremely high metabolic rate. The “Free Radical Theory of Ageing” (FTRA) posits that a high metabolic rate causes mitochondrial heteroplasmy and the progressive ageing phenotype. Contrary to this, bats are the longest-lived order of mammals given their small size and high metabolic rate. To investigate if bats exhibit increased mitochondrial heteroplasmy with age, we performed targeted, deep sequencing of mitogenomes and measured point heteroplasmy in wild, long lived *Myotis myotis*. Blood was sampled from 195 individuals, aged between <1 and at 6+ years old, and whole mitochondria deep-sequenced, with a subset sampled over multiple years. The majority of heteroplasmies were at a low frequency and were transitions. Oxidative mutations were present in only a small number of individuals, suggesting local oxidative stress events. Cohort data showed no significant increase in heteroplasmy with age, while longitudinal data from recaptured individuals showed heteroplasmy is dynamic, and does not increase uniformly over time. We show that bats do not suffer from the predicted, inevitable increase in heteroplasmy as posited by the FRTA, instead heteroplasmy was found to be dynamic, questioning its presumed role as a primary driver of ageing.

## Introduction

The mitochondrion in mammals harbours a ~16.5 kb, circular chromosome, or mitogenome. Across vertebrates the mitogenome shows a largely conserved gene content and synteny, containing 13 protein coding genes, 2 ribosomal RNA genes, 22 transfer RNA genes and a control region. Protein coding genes encode essential subunits of the electron transport chain (ETC) complexes, while the tRNA and rRNA genes encode components of the mitochondrial translation machinery. The mitogenome is maternally inherited, is non-recombining and has a higher mutation rate than the nuclear genome^1,2^. These properties have made it an attractive study locus for phylogenetics, population genetics and forensics. The mitochondria have also been the focus of ageing research since the 1950s due to their central role in the “Free Radical Theory of Ageing”^3^.

The Free Radical Theory of Ageing (FRTA) states, through normal mitochondrial function, reactive oxygen species (ROS) are generated which damage biomolecules leading to mitochondrial dysfunction^3^. ROS are generated when electrons leak from the ETC and reduce oxygen in the absence of hydrogen cations. The mitogenome was once thought to be particularly susceptible to oxidative damage due to its lack of protective histones and close proximity to the ETC, though it is now known that the mtDNA is not naked. Rather, the mtDNA and the TFAM protein compact to form DNA-protein aggregates, called nucleoids, which in mammals typically contain a single mitogenome^2,4,5^. These oxidative mutations become hardcoded in the mitogenome, leading to production of mutated ETC components, and mitochondrial dysfunction. Increased mitochondrial dysfunction gives rise to greater ROS production, which cause greater dysfunction in a “vicious cycle”. This gradual accumulation of heteroplasmy and dysfunctional mitochondria is thought to give rise to the familiar progressive, ageing phenotype^5–7^.

Mitochondrial heteroplasmy is the presence of multiple non-identical mitogenomes in a single individual^8^. Mitogenomes may vary in length or at single nucleotides, referred to as length and point heteroplasmy respectively, where a locus is defined as heteroplasmic if it is heterozygous within an individual and the minor allele frequency (MAF) exceeding some defined threshold. Heteroplasmy plays an important role in mitochondrial disease, cancer and ageing^7,9–11^. Once thought to be extremely rare in human populations, recent next generation sequencing efforts have found extensive heteroplasmy in healthy individuals^12^. These heteroplasmies are predominantly at low frequency, enriched for pathogenic mutations, and show tissue and allele specific patterns^13–15^. As the mitochondrial genome is maternally inherited, studies of mother-child pairs have shown that maternal age at conception is positively correlated with levels of heteroplasmy^16^. More generally, levels of heteroplasmy have been shown to increase with age in humans, and contribute to debilitating age related diseases, as well as developmental disorders such as autism^10,13,15,17^.

The correlated increase in low level heteroplasmies with age in humans seems to support the FRTA. However, human studies have shown a lack of oxidative transversions but instead show transitions are the primary source of mtDNA mutations^13,15,18^. Further, numerous studies have challenged the FRTA. Knock down of key antioxidant defences has been shown to have often contradictory effects on lifespan^19^. The long lived naked mole rat has been found to have higher levels of oxidative damage than other rodents, and has lost important antioxidant enzymes^20,21^. However, these studies have not directly measured mitochondrial DNA damage, and apart from humans, little is known about low level heteroplasmy in mammals. Studies involving mutant mouse models have been used to test the relationship between mitochondrial mutations and ageing. POLG^−/−^ mice express a mitochondrial polymerase with no proof reading activity^22,23^ and so accumulate mitochondrial mutations faster than wild type mice. These mice show a strong progeroid phenotype, supporting a role for mitochondrial mutations in ageing^23,24^. Mammalian studies have been restricted to the control region, often in repeat containing loci, and have not investigated any potential relationship with age^25–32^. Moreover, few comparative studies have taken advantage of recent advances in next generation sequencing technologies. Recently, Rensch *et al*.^1^ used publicly available ChIP-Seq data from 16 species to evaluate patterns of heteroplasmy across the vertebrate phylogeny and concluded that divergent species show similar patterns in frequency and location of heteroplasmy. This study discovered relatively few heteroplasmies, likely due to the high frequency cut off used, and few individuals available for some species. They did not investigate change in heteroplasmy levels with age. To date, next generation sequencing methods have not been used to investigate the relationship between heteroplasmy and age outside of humans.

Chiroptera, the bats, display exceptional longevity for their body size and metabolic rate. Bats are the longest lived order of mammals given their body size, living almost ten times longer than expected^33,34^. The longest lived bat is *Myotis brandtii* (weight ~7 g), which was first captured as an adult and recaptured 41 years later showing negligible signs of senescence^35,36^. Interestingly, bats are the only mammals capable of true powered flight^37,38^. This energy intensive form of locomotion requires an extremely high metabolic rate. During flight the metabolic rate in bats is up to 3 times higher than that of a terrestrial mammal of the same size during exercise^39^. Bats can increase oxygen consumption 20–30 fold during flight, and *M. velifer* has been shown to increase oxygen consumption rate 130 fold^39,40^. According to the FRTA, increased oxygen consumption as a result of flight should lead to increased ROS production, thereby accelerating the ageing process. However, bats seem to defy the FRTA^33,41^. *M. lucifugus* has been shown to produce less ROS per unit of oxygen consumed compared to mammals of a similar size, suggesting *Myotis* bats have increased “mitochondrial efficiency” and have potentially evolved some mechanism to avoid or manage flight-induced oxidative stress^42^.

Heteroplasmy has previously been studied in the long lived *Myotis* genus. Petri *et al*.^26^ studied length and point heteroplasmy in the control region of the *M. myotis* mitogenome. They found high levels of diversity within individuals at a tandem repeat between the tRNA Proline and the conserved sequence blocks of the control region. Sequence diversity at this locus within individuals was similar to diversity between colonies from Germany and Portugal. The length of the repeated array also varied at this locus between and within individuals, with approximately 40% of individuals showing at least two different size arrays. Kerth *et al*.^43^ studied the homologous repeat array in *M. bechsteinii* from Germany and exploited the high levels of sequence and length diversity in this array to study “microgeographic” population structuring in these bats. This locus has been suggested to possibly regulate mitochondrial transcription due to homology with termination-associated sequences leading to a complex secondary structure^32,44^. However, this repeat locus is found only in the Vespertilioninae and Myotinae subfamilies^44,45^, and so cannot apply generally to exceptional longevity across the chiropteran phylogeny. Heteroplasmy has also been studied outside the Vespertilionidae. The distantly related *Rhinolophus sinicus* has was found to exhibit point and length heteroplasmy. As in the studies in vespertilionid bats, heteroplasmy was observed at a tandem repeat in the control region using PCR and cloning based techniques^25^. So far, no study has used next generation sequencing to investigate low frequency heteroplasmy across the whole mitogenome in a long-lived bat species, or tested if heteroplasmy shows any association with age in bats.

To elucidate if bats exhibit increased mitochondrial heteroplasmy with age as predicted by the FRTA, we performed targeted, deep sequencing of mitogenomes and measured point heteroplasmy in wild, long lived *M. myotis* as they age. We report, for the first time, population level characterization of heteroplasmy based on whole mitogenomes from 195 tagged individuals of the long-lived bat, *M. myotis*. Polymerase error likely explains the majority of mutations, with oxidative transversions at a lower rate and concentrated in only a few individuals. Most heteroplasmies are at low frequency, less than 5%. We also sampled and sequenced individuals over multiple years, to assess changes in levels of heteroplasmy at the individual level through time. These unique samples revealed the dynamic nature of heteroplasmy, possibly as a consequence of transient oxidative stress. Contrary to the FRTA, we found that *M. myotis* do not suffer widespread oxidative damage due to flight, instead exhibit acute and transient oxidative stress. We suggest enhanced mitochondrial quality control mechanisms, which may repair or remove damaged mitochondria possibly contributes to the exceptional longevity in *M. myotis*.

## Results

### Sequencing and Quality Control

252 samples were sequenced obtaining a total of 72,111,278 read pairs prior to quality control. After adapter trimming and stringent quality filtering of reads, 56,608,559 (78.5%) of read pairs were retained. Stringent quality control (QC) resulted in loss of more than 20% of raw data. 20 samples were deemed unreliable after testing the effect of PCR duplicate removal. Average coverage of samples ranged from 142 to 7800X. 4 samples with average coverage below 1000X were removed. 12 which failed initial QC tests were re-sequenced, and the same quality control steps as before were performed. 4 re-sequenced samples were retained after quality control. A total of 232 samples were retained for further downstream analysis. The average coverage of the 232 QC passed samples was 3837X, ranging from 1355 to 7800X.

### Sample Demography

As the individuals have been tagged with passive integrated transponder (PIT) tags since 2010, it was possible to identify which samples belonged to a unique individual, and know the age for each individual. For individuals caught first as an adult only a minimum bound for the age was known. The 232 QC passed samples included 88 juveniles (<1 years old), and 143 adults (>1 years old) and one individual for which age could not be reliably assigned (Table 1). The 232 samples represent 195 unique individuals (Table 2), with 21 individuals sampled two, three or four times over consecutive years. All juveniles are 0 years old, with adult samples ranging from 1 years old to at least 7 (hence denoted as 7+). 167 unique individuals aged between 0–5 and a “6+” cohort were used to investigate the relationship between heteroplasmy and age (Table 2).

**Table 1:** Sample demography.

Age	Number of samples, N
Juvenile Samples (0 years old)	88
Adult Samples (>1 year old)	143
1	26
2	22
3	25
4	24
5	3
6+	13
1+, 2+, 3+, 4+, 5+, 7+	30
No age assigned	1

Number of samples assigned to each age cohort.

**Table 2:** Analysed Datasets.

Dataset Title	Description	Number of samples, N
Sequenced Samples	Total number of samples sequenced	252
QC Passed	Samples which passed quality control	232
Unique Individuals	Unique individual bats, recaptures removed	195
Primary	Unique individuals assigned to age cohorts used in model fitting	167
Recaptures	21 individuals caught and sequenced at least twice between 2013 and 2016	56

Description of and number of samples in each datasets used for downstream analyses.

### Simulation Results

9 read sets were generated using GemSIM^46^, ranging from 50 to 25,000X coverage. Each read set was analysed using one of three callers, LoFreq, VarScan or FreeBayes, producing 27 variant call sets, which were given a score between 0 and 1 defined as (Power*Accuracy)*(1-False Positive Rate) (described in full in Material and Methods, Variant Simulation). LoFreq was the best scoring caller (Supp. Fig. 1), and used to call heteroplasmies in all empirical datasets. FreeBayes consistently had the highest power, detecting more than 85% of true variants even at coverages as low 100×, however, FreeBayes also exhibited a false positive rate >5% at all coverages. LoFreq and VarScan had extremely low error rates, with each detecting only one false positive in the 27 variant call sets. LoFreq had a higher score than VarScan at all coverage levels (see Additional File 1).

### Frequency and distribution of heteroplasmies in *M. myotis*

**Figure 1:**
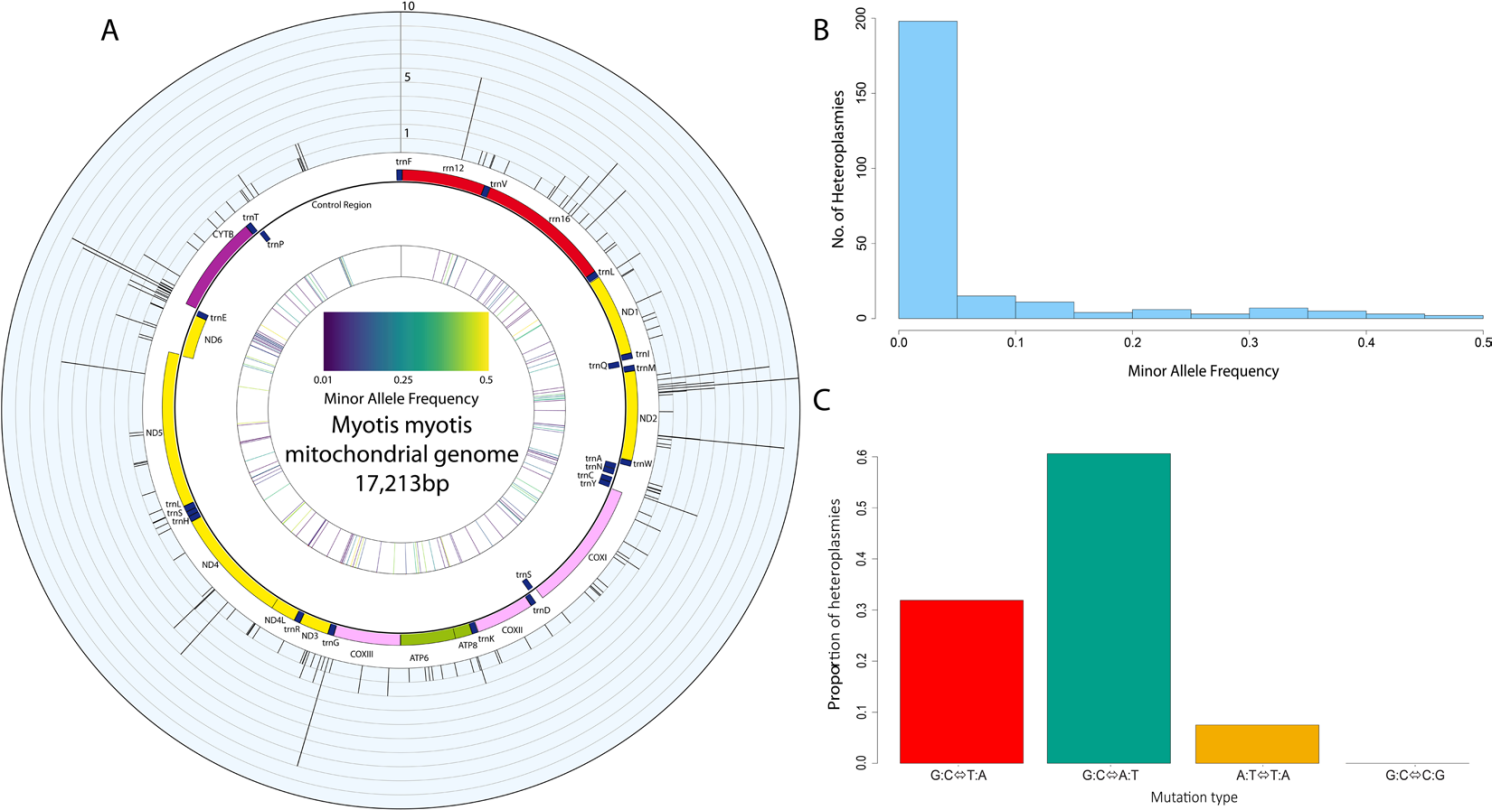
Characteristics of heteroplasmy in *M. myotis*. (**A**) A circos plot of the mitogenome of *M. myotis*. In the outer ring histogram, each bar is one of the 143 unique sites which were heteroplasmic in the population, with the height representing the number of individuals sharing the site. The middle ring is a schematic of the mitogenome generated with OGDRAW^87^. The inner most ring is a heat plot showing the average minor allele frequency at each heteroplasmic site, with low frequencies in purple and high frequencies in yellow. (**B**) Histogram of minor allele frequencies of 254 heteroplasmies discovered. The vast majority of heteroplasmies were at a frequency below 5%. (**C**) Bar chart showing the proportion of mutations attributable to one of four transition or transversion classes. The transition transversion ratio (Ts/Tv) for *M. myotis* was 1.8. Oxidative mutations, G:C⇔T:A transversions, were at a higher level than reported previously in humans.

From 232 samples, 254 point heteroplasmies, across 143 sites in the mitogenome were discovered. Sites were called as heteroplasmic if they were heterozygous with an intra-individual minor allele frequency (MAF) greater than 1%, Fig. 1A. The frequency of minor alleles was strongly skewed toward low frequency variants. Figure 1B shows a histogram of number of heteroplasmies by MAF for all 254 heteroplasmies. The vast majority, 77.6%, of heteroplasmies were below 5% MAF. The low MAF found for the majority of heteroplasmies suggests these are likely somatic. However, it is not possible to distinguish between somatic and inherited heteroplasmies given the samples are from only one tissue and maternal relationships between individuals are unknown.

210 heteroplasmies were collected from 195 unique individuals, using the most recent sample from recaptured individuals. The difference between the frequency distribution of coding and non-coding heteroplasmies was approaching significance (Kolmogorov-Smirnov (KS) Test, p = 0.054) with non-coding heteroplasmies tending to be at lower frequencies. Closer investigation of coding heteroplasmies found nonsynonymous heteroplasmies were at a lower frequency on average than synonymous (Mann-Whitney U-test, p = 0.021 Supp. Fig. 2A).

This also drove the difference observed between coding and non-coding heteroplasmies, as a significant difference was found between the frequency distributions of synonymous and non-coding heteroplasmies, but not between nonsynonymous and non-coding heteroplasmies (KS Test, p = 0.006 and p = 0.374 respectively) (Supp. Fig. 2A). There was also a difference in the frequency of heteroplasmies depending on the codon position with heteroplasmies at the second codon position at a lower frequency than position 1 or 3, which showed no difference to each other (KS Test, p = 0.004, p = 0.005, p = 0.411 respectively) (Supp. Fig. 2B). To further explore the effect of heteroplasmies, all nonsynonymous heteroplasmies, excluding nonsense mutations, were scored as “Deleterious” or “Neutral”. Of the 62 nonsynonymous sites scored, 42 were found to be deleterious and 20 neutral. Deleterious heteroplasmies were at significantly lower frequency than neutral heteroplasmies (KS Test, p = 0.012).

Heteroplasmic sites were spread relatively evenly across the mitogenome. The numbers of heteroplasmic *versus* homoplasmic sites were used to construct a contingency table for each mitochondrial partition. Dividing the mitogenome into tRNA, rRNA, protein coding and non-coding regions, showed enrichment for heteroplasmic sites in tRNA genes (*Χ*^2^ (1, N = 143) > 10.828, p < 0.001). Analysis of individual genes revealed *ND2*, *trnaE* and *trnaN* genes were enriched for heteroplasmic sites (*Χ*^2^ (1, N = 143) > 3.841, p < 0.05). The majority of sites, 72.7%, were only observed as heteroplasmic in one individual (Fig. 1A), hereafter called “private” heteroplasmies as opposed to “shared”. While shared heteroplasmies are more likely to be inherited, and shared between maternally related individuals, particular sites within the mitogenome show higher rates of heteroplasmy in unrelated humans^13^, we thus make no assumption as to whether shared and private mutations are somatic or inherited. Private and shared heteroplasmies showed significantly different frequency spectra (KS Test, p = 0.005) with private heteroplasmies at lower average frequency. Private and shared heteroplasmic sites showed similar proportions of synonymous and nonsynonymous mutations and no bias toward coding versus non-coding sites (*Χ*^2^ (1, N = 143) < 2.706, p > 0.1 in all comparisons).

### Transitions are the primary source of heteroplasmies

210 heteroplasmies from 195 unique individuals were classified into 4 classes, 1 transition class (G:C ↔ A:T) and 3 transversion classes (G:C ↔ T:A, A:T ↔ T:A and G:C ↔ C:G), as shown in Fig. 1C. Transitions were the primary class of heteroplasmy at 60.6%. This was followed by G:C ↔ T:A transversions at 31.9%, which are characteristic of oxidative stress events. The remaining 7.5% of mutations were A:T ↔ T:A transversions. *M. myotis* showed a small transition to transversion (Ts/Tv) ratio, with a Ts/Tv of 1.8. Only 38 samples (16.4%) showed evidence of oxidative transversions, of which 33 were unique individuals. The 5 samples containing the highest number of oxidative transversions accounted for 37% of these transversions (30 of 81).

### Association between heteroplasmy and age

**Figure 2:**
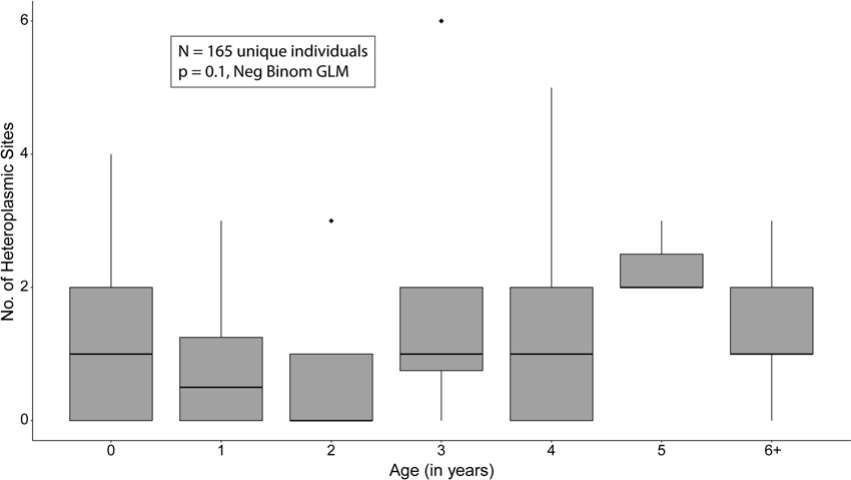
Heteroplasmy is not significantly associated with age in *Myotis myotis*. Boxplots of the distribution of heteroplasmy counts in each age cohort used for age analysis. No significant association was found between age and heteroplasmy using a negative binomial generalized linear model, after the removal of unduly influential points.

To test if the number of heteroplasmic sites within an individual showed an association with age we fitted a generalised linear mixed model. The model incorporated age as the sole fixed effect and included sequencing batch as a random effect, with the count of heteroplasmic sites as the negative binomial distributed response variable. Sequencing batch was included as samples from different batches exhibited significantly different numbers of heteroplasmies (Kruskal-Wallis test, p = 0.007). However, the magnitude of the difference in means was small, with a maximum difference of 1.7 heteroplasmies between two batches. Furthermore, batch as a random effect had an estimated variance of 0, indicating the model was degenerate. As such, the simpler generalized linear model with age as the sole fixed effect was retained for further analysis (Table 3). Two samples within the 6+ cohort were found to be unduly influential points using Cook’s Statistic (Supp. Fig. 3A,B). As such they were removed and the model fit again. With these two samples removed no significant association with age was found (ANOVA, p = 0.1), as shown in Fig. 2 and Table 3. Bootstrapping of the three fitted models also showed a decrease in the standard error and bias for the model estimates with the outliers removed, Table 3. Further, the two outlier samples contained primarily oxidative mutations, one of which contains the highest number of oxidative mutations in our data. To investigate any bias that may be introduced by choosing the most recent sample from recaptured individuals, we produced 1000 permutations in which the sample from recaptured individuals of usable age was chosen at random, to produce a similar dataset of 165 unique individuals. 19.8% of samplings found a significant association between age and number of heteroplasmies (Supp. Fig. 4A), with an average slope 0.0969 and ranging from 0.0815 to 0.1237 (Supp. Fig. 4B).

**Table 3:** Modelling the relationship between age and heteroplasmy.

Dataset	Model	N	Fixed Effect	Random Effect	AICc	Estimate	Bootstrap Std. Error	Bootstrap Bias	p-value
Primary	GLMM	167	Intercept Age	Batch	482.09	−0.1288 **0.1289**	0.1383 0.0575	0.0221 0.0152	0.2809 **0.0017**
Primary	GLM	167	Intercept Age	NA	479.99	−0.1288 **0.1289**	0.1121 0.0465	−0.0094 −0.0028	0.2836 **0.0021**
Influential points removed	GLM	165	Intercept Age	NA	456.80	−0.0843 0.0699	0.1094 0.0384	−0.0059 0.0002	0.4550 0.1000

Regression analyses revealed a significant association with age, but this was due to the presence of two unduly influential points. Bias and standard error were estimated from 1000 parametric bootstrap replicates.

### Longitudinal analysis of heteroplasmy

**Figure 3:**
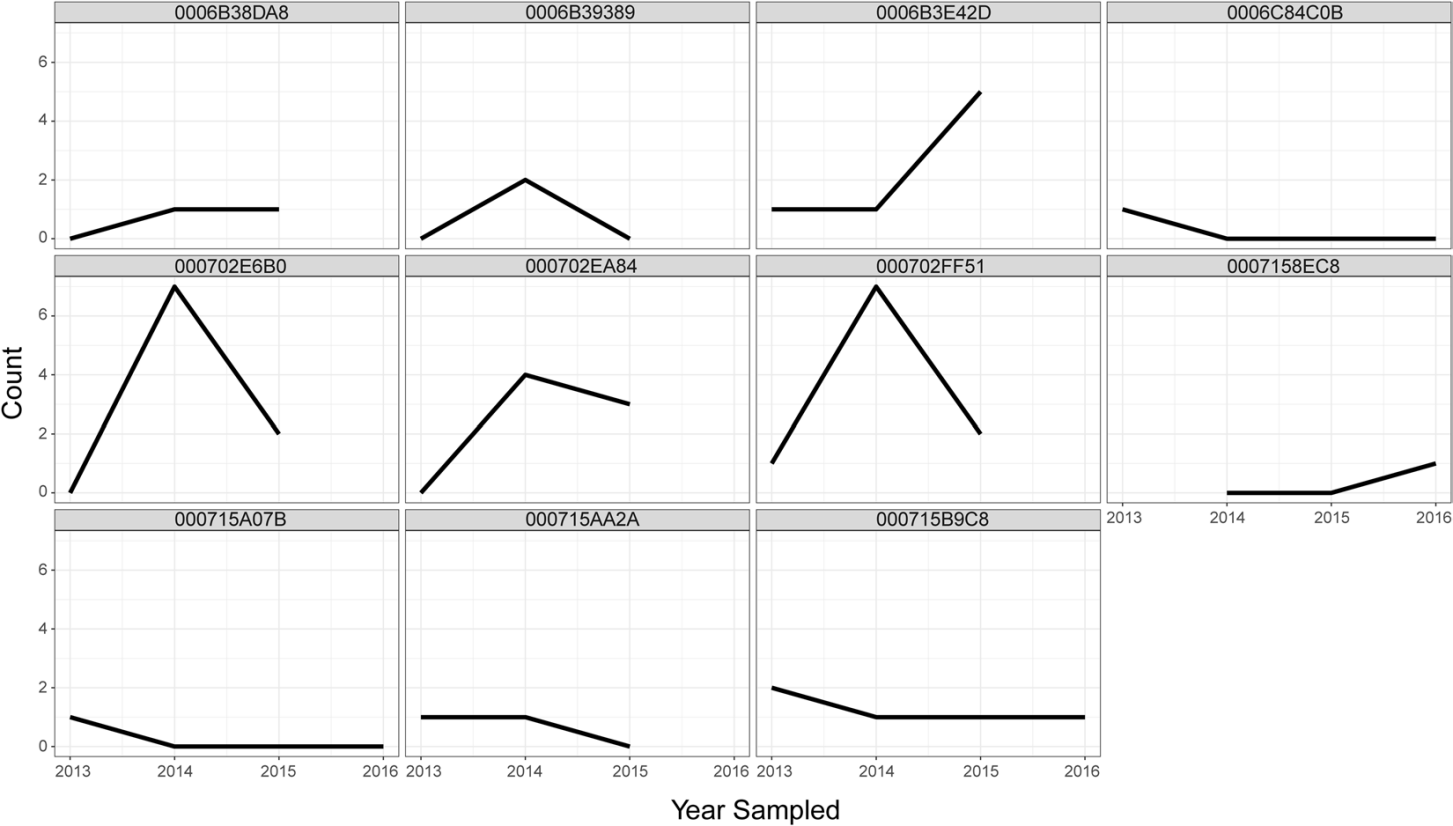
Plots of heteroplasmy count against year sampled for 11 individuals. Heteroplasmy counts through time for 11 individual bats which were sampled 3 or 4 times. Numbers above each plot indicate PIT number used to identify each individual. Some individuals were extremely consistent while others increased sharply, always followed by a decrease where subsequent samples were available.

From the 27 individuals sampled more than once, we were able to obtain samples from 21 individuals from at least two time-points, which passed quality control. 3 individuals were sampled at 4 time-points, 8 individuals sampled at 3 time-points and 10 individuals at 2 time-points, individuals sampled at 3 or 4 time-points are shown in Fig. 3. The mean change in number of heteroplasmies between two consecutive years across individuals was 0.303 (standard deviation ± 2.52). Of the 33 consecutive intervals (e.g. 2013 to 2014 or 2014 to 2015 etc.) 15 showed no change, 10 showed a decrease and 8 showed an increase (Supp. Fig. 5). Not all heteroplasmies were shared between time-points. 14.7% of heteroplasmies were observed in at least two time-points, with 13% shared between all time-points for an individual. Shared sites ranged in frequency from 43.36% to as low as 1.02%. Some individuals were extremely consistent over all time points. For example, individual 000715B9C8, shown in Fig. 3, initially showed 2 heteroplasmies, but subsequently lost one. The remaining heteroplasmy was at a low frequency but was found at all 4 time-points and had an extremely consistent frequency, range_maf_ 1.02–1.83%. However, some individuals were more dynamic. These “spikes” in the number of heteroplasmy was normally followed by a marked decrease the following year. Two bats, 000702E6B0 and 000702FF51 (also shown in Fig. 3), showed spikes and subsequent loss, though they retained a subset of the mutations acquired during the spike. Four individuals showing spikes in the number of heteroplasmy, with an increase of 4 or more sites between two subsequent years, were previously found to have some of the highest levels of oxidative mutations. Interestingly, bat 000702EA84 showed a spike in heteroplasmy with little recovery the following year. Instead, this bat may have experienced oxidative stress events during two consecutive field seasons.

## Discussion

Comparative genomics has emerged as a powerful new tool in ageing research^33,47^. However, though mitochondrial dysfunction and heteroplasmy have been long implicated in ageing, there remains a paucity of data from non-human or non-model organisms to associate increased heteroplasmy as a mechanism underpinning the progressive, ageing phenotype. To this end, for the first time we have deep sequenced, to an average depth of >3500X, whole mitogenomes from 195 *M. myotis* individuals, and investigated the association between heteroplasmy and age in these long-lived bats. After outlier removal, we found no evidence for an increase in heteroplasmy with age. We also found little to no evidence to support the Free Radical Theory of Ageing, finding no chronic increase in oxidative mutations with age. Through unique, longitudinal sampling we found bats may experience local oxidative stress events followed by removal of the majority of mutations. Nonsynonymous, protein coding mutations were also at a significantly lower frequency than synonymous, and those with predicted deleterious effects were lower again. Together our results suggest *M. myotis* may remove deleterious mitochondrial DNA mutations, preventing their expansion, to maintain mitochondrial homeostasis, and possibly promoting longevity. This hypothesis will need further investigation.

The FRTA posits oxidative mutations in the mitogenome should drive mitochondrial dysfunction^3^. However, many studies have shown a dearth of oxidative mutations in mitochondrial DNA^48^. Studies in model and long-lived non-model organisms have also led many to question this central tenets of the FRTA^19,48–50^. We found little support for the FRTA in *M. myotis*. Transitions, not oxidative transversions, are the primary source of mutation. Transitions are likely due to errors during DNA replication or spontaneous deamination of nucleosides, rather than damage accrued by oxidative stress^4,7^. Still, the proportion of heteroplasmies owing to oxidative transversions in *M. myotis* mitochondrial DNA is much higher than reported in humans^13,15^. However, the majority of these oxidative mutations were concentrated in only a few individuals. These likely represent local oxidative stress events, rather than a population wide phenomenon. The longitudinal data supports this interpretation as two individuals exhibiting high numbers of oxidative transversions, and some of the highest levels of heteroplasmy, were also sampled the following year (individuals 000702E6B0 and 000702FF51, Fig. 3). They had cleared or repaired the majority of the oxidative damage, though each retained at least one of the heteroplasmies from the previous year. We suggest *M. myotis* experience local, transient oxidative stress followed by return to homeostasis and removal or repair of oxidative lesions.

As oxidative damage is concentrated, and transient, within a small proportion of individuals, it is unlikely to be the ultimate driver of ageing, as posited in the FRTA. It also seems unlikely that flight is the cause of this oxidative stress, as it should then be ubiquitous across the population rather than concentrated in few individuals. There are multiple other explanations for the transient oxidative damage observed in only some individuals, including infection and recent reproductive effort^51–53^.

In recent years, the role of ROS as essential signalling molecules in immune cells has become clear. *In vitro*, macrophages recruit mitochondria to the phagosome where promotion of H_2_O_2_ production contributes to the control of *Salmonella* infection^53^. Activation of the NLRP3 inflammasome has also been shown to promote ROS production^52^. It has been suggested that bats have a constitutively active innate immune system, possibly explaining their unique ability to act as reservoirs for diverse viruses, such as SARS, which can be pathogenic in humans and other mammals^54–57^. The constitutive expression of interferon or the presence of circulating viruses in bats may lead to acute levels of ROS production in bats enabling them to quickly mediate infection^58,59^. The long lived bat species, *Carollia perspicillata*, has also been shown to experience increased oxidative stress during simulated bacterial infection^60^. Potentially, individuals showing increased oxidative damage were experiencing immune related oxidative stress at the time of sampling, although this remains to be tested. This may also suggest that spikes in oxidative transversions are also a blood specific phenomenon; future studies incorporating different tissues may shed light on the role and effect of ROS in the bat immune system.

A second possibility is reproductive effort. It has been shown that reproductive effort increases oxidative stress in free living animals, though not unequivocally^51^. As the *M. myotis* sampled over the course of the study were caught at maternity roosts, most females will have recently given birth to a pup, and may still be lactating. Under the disposable soma theory of ageing, reallocation of resources from self-maintenance to reproductive effort leads to increased damage of somatic tissue during reproductive phases, with unrepaired damage accumulating with time^51,61^. The spikes in oxidative damage followed by recovery as observed in our longitudinal sampling, showed while most oxidative damage observed during the spike were eventually lost or removed, some mutations persisted, and could be detected the following year. Further longitudinal sampling, or sampling at different life history phases may help elucidate the cause of oxidative stress in these bats.

The relationship between age and heteroplasmy was unclear in bats. A positive correlation with age seen in our data was driven by two samples. These samples were clear outliers with a large effect on our regression. Removal of these samples also removed the association with age. The outliers were both in the oldest cohort and had two of the highest levels of heteroplasmy, primarily due to oxidative mutations. It seems likely these individuals may have been experiencing acute oxidative stress, and may have removed the majority of these oxidative mutations in following years, as seen in other individuals. However, this remains speculative as we have yet to re-sample these two individuals. Further, the observed dynamic nature of heteroplasmy questions the direct contribution of heteroplasmy to ageing in this species. However, as our study population has only been tagged since 2010 we have only sampled a small portion of the life span of these bats, which can live up to 37.1 years. It is possible accumulation of heteroplasmies is not detectable over this time scale, therefore further longitudinal analyses are required to sample individuals at known maximum age.

The question remains, how do bats maintain their efficient mitochondria, and how might this lead to an increased lifespan? Our data shows no support for flight induced oxidative damage accumulating in *M. myotis*. However, oxidative stress is observed in numerous individuals, and those with samples from subsequent time-points suggest possible removal of the majority of mutations, suggesting mitochondrial stress followed by repair or removal of damaged mitochondria. Further, nonsynonymous mutations were at significantly lower frequency than synonymous, suggesting purifying selection acting on these heteroplasmies. Nonsynonymous sites predicted to be deleterious were even lower in frequency again than those with little or no functional impact. This suggests *M. myotis* may prevent expansion of deleterious, nonsynonymous mutations which has been shown to contribute to the ageing phenotype in human and mouse models^23,62^. This may further contribute to the maintenance of mitochondrial function with age.

There are multiple mechanisms by which *M. myotis* may remove and prevent expansion of mitochondrial mutations. One such mechanism is through direct repair of DNA damage. DNA repair genes and pathways have been shown to be evolving or expressed differently in bats compared to other mammals^63–65^, however, many of these pathways are not active in the mitochondria. Base excision repair (BER) is the primary method of DNA repair in mitochondria. While double strand break repair operates in mitochondria, the mechanisms have not been fully characterised^4^. Another mechanism by which *M. myotis* may remove oxidative damage is through mitophagy. Mitophagy is a form of autophagy through which cells remove dysfunctional mitochondria. Inhibition of autophagy, and so mitophagy, by knockdown of autophagosome genes has also been shown to lead to increased ROS production^48^. Mitochondria derived ROS have also been shown to promote autophagy during starvation, through oxidation and inactivation of the ATG4 protein^66^. Long lived bats have also been shown to have increased macroautophagy activity in comparison to a short-lived, phylogenetically close sister taxa. Interestingly, other homeostatic mechanisms such as chaperone protein levels and proteasome activity, showed no difference in long and short-lived bats^67^. This suggests long-lived bats rely on autophagy for maintaining homeostasis. Indeed, previous studies have shown that autophagy genes are upregulated in the blood of *M. myotis* compared to other mammals^65^. Coupled with our results, this suggests *M. myotis* may possess stringent mitochondrial quality control to prevent accumulation and expansion of deleterious mutations, contrary to what has been shown in humans^13,15,16^. Continued longitudinal sampling and experimental validation are necessary to determine if these mechanisms truly prevent or limit accumulation of heteroplasmies with age in *M. myotis*. Also, further comparative studies are needed to ascertain if this ‘maintenance phenotype’ is common across Chiroptera and amongst other long lived species.

In conclusion, we found little support for the long standing “Free Radical Theory of Ageing” in the exceptionally long-lived bat, *M. myotis*. Instead, oxidative mutations were not the primary source of mutations and most seemed to be transient, arising from local oxidative stress events before disappearing. We did not find that accumulation of heteroplasmic sites in the mitogenome increases with age in these bats, however, we have only analysed a small portion of the lifespan of this species. Unique longitudinal sampling revealed the dynamic nature of heteroplasmy, with some bats gaining or losing up to 6 heteroplasmies between consecutive years. These drastic shifts in heteroplasmy suggest increases in heteroplasmy may not only be tolerable but removable in *M. myotis*. We propose that stringent mitochondrial quality control mechanisms drives mitochondrial health, potentially contributing to the exceptional longevity of *M. myotis*.

## Methods

### Capture and Sampling

All procedures were carried out in accordance with the ethical guidelines and permits delivered by University College Dublin and the Préfet du Morbihan respectively. *M. myotis* were sampled in western France in 2013, 2014, 2015 and 2016 (detailed in Table 4) as previously described^3^.

**Table 4:** Colony location and sample numbers.

Colony	Latitude	Longitude	No. Samples
Béganne	N47°35′	W2°14′	112
Noyal-Muzillac	N47°35	W2°27	20
Férel	N47°28′	W2°20′	78
La Roche-Bernard	N47°31′	W2°17′	32
Limerzel	N47°38′	W2°21′	10

Latitude and longitude of 5 sampling colonies, and the total number of sequenced samples originating from each.

### DNA Extraction

Prior to DNA extraction all blood samples had RNA extracted using the RNAzol BD for blood kit (catalogue number RB 192, Molecular Research Centre, Inc.) using the manufacturers protocol with minor modification as previously described^68^. Post extraction of the RNA containing layer, the phenol phase/interphase, was placed at −80 °C. DNA was extracted from the phenol phase/interphase using the protocol detailed in the RNAzol BD kit manual with minor modifications. Briefly, for each original volume of blood, the DNA extraction buffer was prepared with 1 volume of 4 M guanidine thiocynate solution, 0.1 volumes of 3 M sodium hydroxide and 0.005 volumes of polyacryl carrier. Samples were allowed to thaw in the DNA extraction buffer and then were vortexed vigorously for 30 s. Samples were incubated at room temperature (RT) for 10 minutes, then vortexed for 30 s. 0.2 volumes of chloroform was added to each sample, briefly vortexed, and centrifuged at 12000 rpm for 20 mins at RT. The upper fraction was extracted, and extracted once more with an equal volume of chloroform where necessary, to produce a clear, aqueous DNA phase. DNA was precipitated overnight at −20 °C in an equal volume of isopropanol. DNA was pelleted by centrifugation at 12000 rpm for 20 mins at 4 °C. DNA pellets were washed twice with 75% ethanol and then resuspended in 30 µl nuclease free water.

### mtDNA Enrichment and Sequencing

The whole mitogenome of *M. myotis* was amplified and sequenced using the primers, and sequencing protocols outlined previously^69^. Briefly, samples were enriched for mitochondrial sequences using long range PCR, in two overlapping ~10 kb fragments. Both amplicons were successfully amplified for 252 samples. The two amplicons for each of the 252 samples were purified by vacuum filtration (MilliporeHTS Filtration Plate, cat. no. MAVM0960R), quantified using a nanodrop spectrophotometer and then pooled in equimolar amounts. Pooled amplicons were used to produce sequencing libraries using the Nextera XT preparation kit as per the manufacturer’s instructions. Varying numbers of samples were multiplexed and loaded onto an Illumina MiSeq.

### Quality Control

Raw reads were initially processed using a stringent quality control pipeline prior to variant calling. Samples were trimmed of Illumina and Nextera adapter sequences using Cutadapt (v1.8.3)^70^ and The NGS QC Toolkit^71^ was used to filter out reads for which 80% of bases had quality scores less than Q30. Due to extreme sequence depths at small target regions in resequencing studies, it is possible that inserts will begin and end at the same position due to chance, called sampling coincidence. Reads from such inserts will be falsely identified as PCR duplicates. However, not removing true PCR duplicates may affect the accuracy of variant calling. In order to remove any potential bias introduced by duplicate removal or retention, we ran the heteroplasmy detection pipeline with duplicates present and removed. As some samples showed extremely high levels of variation between treatments we used the interquartile range to identify and remove outliers. An outlier was defined as any value for which the count value was 2 interquartile ranges below the first quartile or above the third quartile. The mean and standard deviation of these filtered counts was then calculated. A cut-off value of two standard deviations from the mean was rounded down to the nearest integer value. Samples with a difference in counts between the two treatments, which was greater than this cut-off were deemed unreliable and removed from downstream analysis. Finally, average coverage was calculated for each sample using the depth command in samtools (v0.1.19)^72^. As all variant callers exhibited power below 80% at coverage below 1000X (Fig. 2), samples with an average coverage below this threshold were removed.

### Heteroplasmy Detection Pipeline

The first and last 500 bp of the mitogenome were copied to the opposite ends, to extend the reference and account for circularity of the mitogenome. Reads were mapped against the extended *M. myotis* mitogenome using the BWA-MEM^73^ algorithm. Prior to variant calling BAM files were processed using Picard Tools (v1.90)^74^ and the Genome Analysis Tool Kit (GATK)^75^. Briefly, SAM files were sorted and read groups added using Picard Tools. SAM files were then converted to BAM files and indexed. Prior to base quality recalibration a set of raw variants was produced using the chosen low frequency variant caller, either LoFreq Star^76^ or VarScan 2^77^. These raw variants were used as the “known sites” necessary for the BQSR walker in the GATK. As no databases of variants exist for most non-model organisms, these raw variants provide an *ad hoc* solution. Final heteroplasmies were called on the recalibrated data again using the caller of choice. A third variant caller, FreeBayes^78^, does not require recalibrated or realigned data, and so these steps were omitted when FreeBayes was used. For the empirical data, variants with a minor allele frequency greater than 1% and at sites with greater than 1000X coverage were retained. These variants were then annotated, removing variants in primer binding sites and the two Myotinae repeats in the control region. An illustration of the bioinformatic workflow for a single sample from quality processing through to annotated variants is depicted in Supp. Fig. 6. GNU Parallel (v20170122)^79^ was used to parallelise the mapping and processing of SAM and BAM files, markedly decreasing computational time. The number of samples to be run in parallel can be defined by the user.

### Variant Simulation

In order to gauge the sensitivity, accuracy and false positive rate of our bioinformatic pipeline, known variants and Illumina sequence data were generated *in-silico* using the GemSIM package (v 1.5)^46^. Sequence reads in FASTQ format from a reference individual (MMY104, used to generate the reference mitogenome for this species) were mapped to the previously published *M. myotis* mitogenome^69^ using the BWA-MEM algorithm, producing a SAM file which was used to produce a general error model using the GemErr.py module. The reference mitogenome was used to generate haplotype information for variant simulation with the GemHap.py module. In total 500 known variants were simulated, in sets of 100 at frequencies of 1%, 4%, 7%, 10% and 15%. 7 sites were found in two datasets and were excluded from the analysis, for a total of 486 heteroplasmic sites out of 17,211. The reference mitogenome, error profile and haplotype information were used to generate 250 bp paired end Illumina reads. Datasets were simulated at 50X, 100X, 250X, 500X, 1000X, 2500X, 5000X, 10,000X and 25,000X coverage. Predicted variant positions were checked against the known set. Power (proportion of true sites called), false positive rate (proportion of erroneous sites called) and accuracy (proportion of true sites with predicted frequency within ±0.01 of the true frequency) were calculated on each dataset for each caller, FreeBayes, LoFreq and VarScan2 (see Heteroplasmy Detection Pipeline). Finally a score between 0 and 1 was assigned to each caller defined as (Power*Accuracy)*(1-False Positive Rate). All variants called from empirical samples used the best performing caller.

### Characterising heteroplasmy in *M. myotis*

All filtered and annotated heteroplasmies were collected and manually inspected. Heteroplasmies were binned into several overlapping classes: protein-coding, synonymous, nonsynonymous, non-protein-coding, tRNA, rRNA, non-coding, and by each mitogenome feature. Nonsynonymous mutations were further binned for possible effect as “Deleterious” or “Neutral” based on the PROVEAN algorithm as implemented on the dedicated web server. Default cut-offs were used such that a mutation was binned as “Deleterious” if the computed PROVEAN Score was greater than 2.5 or less than −2.5^80,81^. Frequency distributions for each bin were compared using a two tailed non-parametric Kolmogorov-Smirnov Test or Mann-Whitney U-test. The number of unique heteroplasmic sites in a bin was tested for enrichment against the remainder of mitogenome sites analysed (length of mitogenome – primer regions – number of sites covered by bin), using Pearson’s Chi-Square test.

### Association between heteroplasmy and age

A primary dataset of n = 167 was constructed from unique individuals of known age and an oldest cohort, divided into cohorts from 0 to 6+, Tables 1 and 2, taking the most recent sample of an individual had the bat been sampled multiple times. The number of heteroplasmies for samples was compared between sequencing batches using Kruskal-Wallis test. The primary dataset was used to test for a significant association between number of heteroplasmic sites and age by fitting a generalised liner mixed model (GLMM) in R (v3.3.3) using the “lme4” package^82^ with age as the only fixed effect and incorporating sequencing batch as a random effect, and number of heteroplasmic sites as the response variable modelled under a negative binomial distribution. A negative binomial distribution was used due the presence of overdispersion in the data making a Poisson distribution unsuitable. Subsequently a generalised linear model (GLM) was fit using the “MASS” package^83^ with age as the sole explanatory variable. The “boot” package^84,85^ in R (v3.3.3) was used to diagnose samples which may be unduly influential points using the Cook Statistic^86^. Influential points were removed and the model was fitted again. 1000 bootstrap replicates were generated for each fitted model by randomly sampling with replacement from the datasets using the “boot” package in R. The standard error and bias for the Intercept and Age estimates were recorded. To investigate any bias that may be introduced by manually selecting a sample from recaptured individuals, we performed 1000 samplings in which the sample from recaptured individuals of usable age was chosen at random, to produce 1000 datasets (of n = 165) and the model fit again.

### Longitudinal Analysis

*M. myotis* show natal philopatry, returning to the same roost or broad area to give birth each year. As such, it was possible to recapture the same individuals almost every year. 27 individuals with samples from at least two time-points were sequenced; samples that passed quality control were retained, for a final set of 56 samples from 21 individuals (Tables 2 and 5). We calculated the mean change between any two consecutive years over all individuals, excluding 2 which were sampled non-consecutively. We also calculated the proportion of shared heteroplasmies between any two time-points, and those shared between all time-points.

**Table 5:** Recaptured individuals and associated samples.

Number of times sampled	Number of Individuals	Number of Samples
4	3	12
3	8	24
2	10	20

Number of individuals sampled 4, 3 or 2 times between 2013 and 2016. The final number of samples shown is for QC passed samples. Only these samples were used in recapture analysis.

### Ethics approval

All procedures were carried out in accordance with ethics approval issued by University College Dublin to Prof. Emma Teeling (AERC_1338_Teeling) and in accordance with Arrêté préfectoral permits issued by the Préfet de Morbihan to Frédéric Touzalin and Sébastien J. Puechmaille.

## Electronic supplementary material

Supplementary Figures

Supplementary Dataset 1

## Data Availability

Annotated variants and sample information used for statistical analyses are available within Additional File 1. The scripts and binaries for all dependencies needed to run the 3 ML pipeline are available at https://github.com/jebbd/3 ML. Sequence data generated during the course of the project from empirical datasets are available via the Sequence Read Archive, SRP158182.
