# Supplementary material for: Population level mitogenomics of long-lived bats reveals dynamic heteroplasmy and challenges the Free Radical Theory of Ageing
Source: Sci Rep. 2018 Sep 11;8:13634. doi: 10.1038/s41598-018-31093-2 (PMC6134106; doi:10.1038/s41598-018-31093-2)
Supplement: Supplementary file 1 — Supplementary Figures [file 41598_2018_31093_MOESM1_ESM.pdf]

Population level mitogenomics of long-lived bats reveals dynamic heteroplasmy and challenges the Free Radical Theory of Ageing

David Jebb[1], Nicole M. Foley[1], Conor V. Whelan[1], Frédéric Touzalin[3], Sebastien J. Puechmaille[1,2], Emma C. Teeling[1]*

[1]School of Biology and Environmental Science, University College Dublin, Belfield, Dublin 4, Ireland

[2]Applied Zoology and Conservation, Zoological Institute, Greifswald University, Greifswald, Germany

[3]Laboratoire Evolution et Diversité Biologique, Université Toulouse 3, Paul Sabatier 31062 Toulouse Cedex 09, France.

*Corresponding author: emma.teeling@ucd.ie

Supplementary Figures

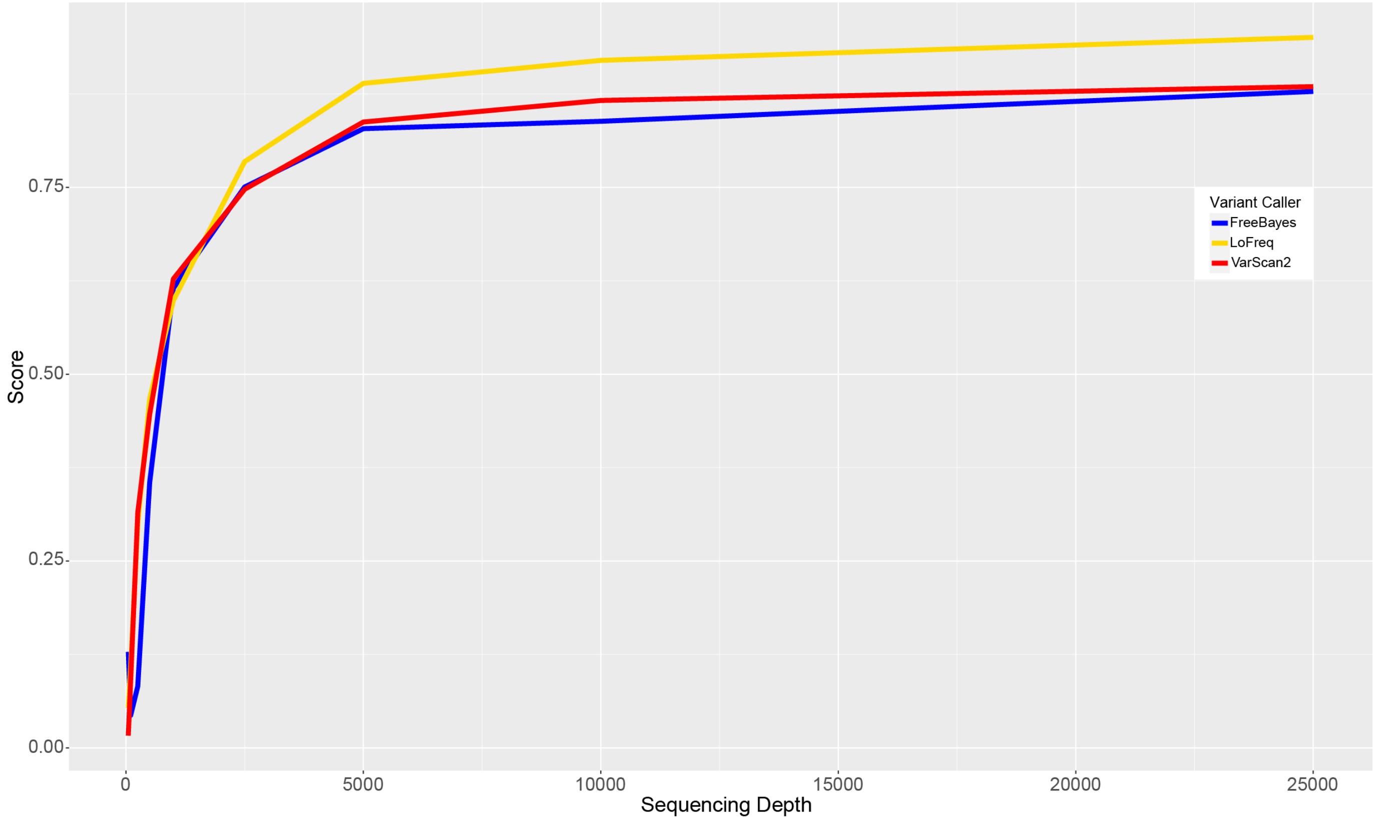

Supplementary Figure S1: Results from simulated datasets using different variant callers. Nine read sets were generated *in silico* using GemSIM and the *M. myotis* mitochondrial genome, containing 500 known variants. The heteroplasmy detection pipeline was run on each dataset three times, using a different variant caller, with or without INDEL realignment and base quality recalibration as appropriate. The 27 resulting variant sets were compared to the known set for power, accuracy and false positive rate. A score was given to each set as (Power*Accuracy)*(1-False Positive Rate), and plotted against the expected coverage for the set. LoFreq was the best performing caller and was used for all variant calling on real data.

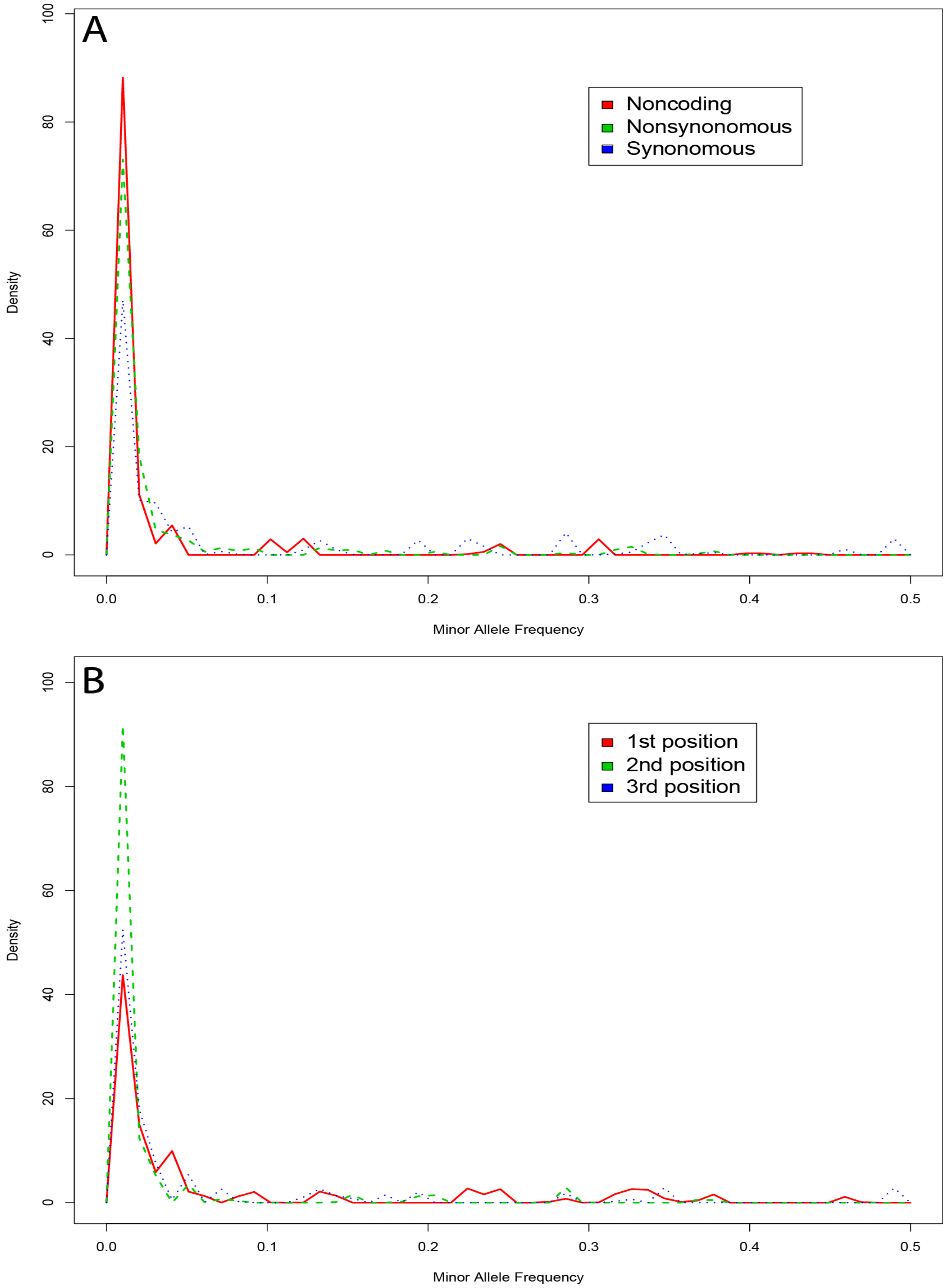

Supplementary Figure S2: Frequency distributions and comparisons for different classes of heteroplasmies. A) Density plots of minor allele frequencies for noncoding, nonsynonymous coding and synonymous coding mutations. **B)** Density plots of minor allele frequencies for heteroplasmies at the first, second and third codon position.

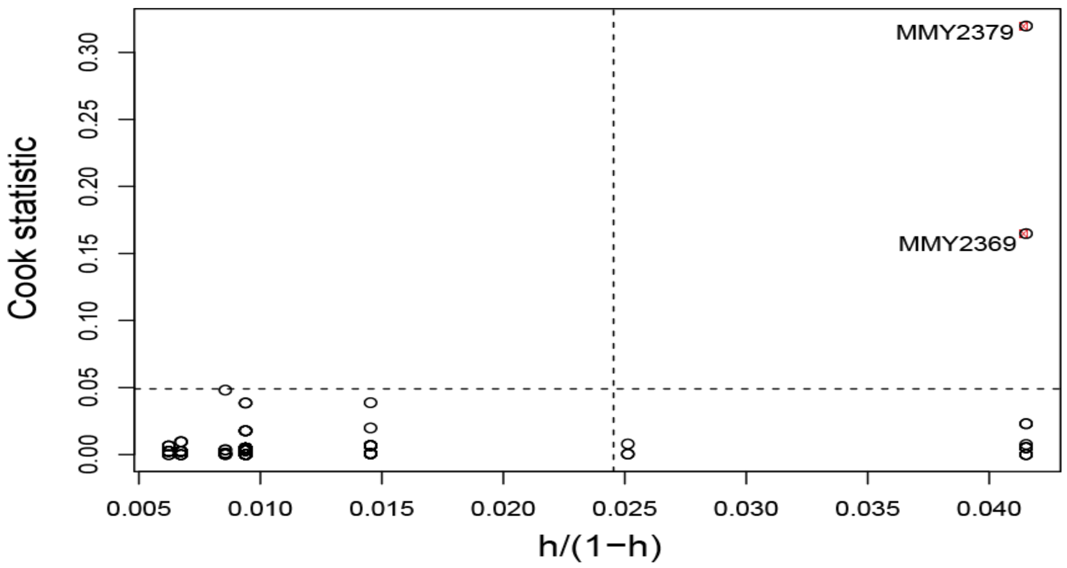

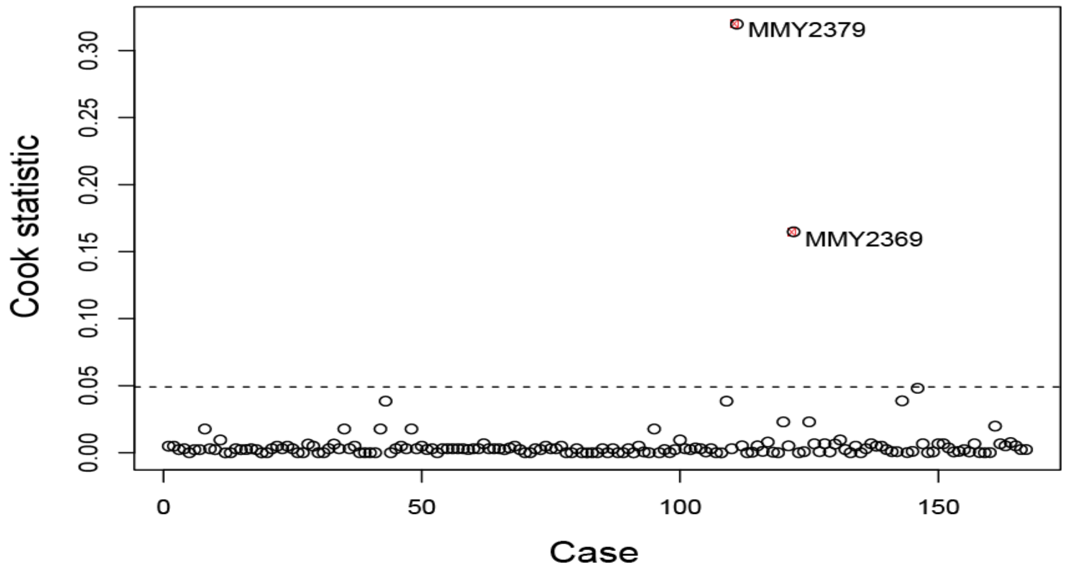

Supplementary Figure S3: Cook's Statistic predicting influential points in the Primary dataset. A) A plot of Cook's statistics versus the standardised leverages for each point. The horizontal line is at 8/(n-2p) and the vertical line is at 2p/(n-2p) where n is the number of observations and p is the number of parameters estimated. Points above the horizontal line have a high influence on the model, and those to the right of the vertical line have a high leverage. The two outliers previously shown in Figure 4 are again indicated in red, with both above the horizontal and right of the vertical lines. **B)** A plot of the Cook's statistics for each observation, order as they appear in the dataset. Again the two highly influential observations are coloured in red.

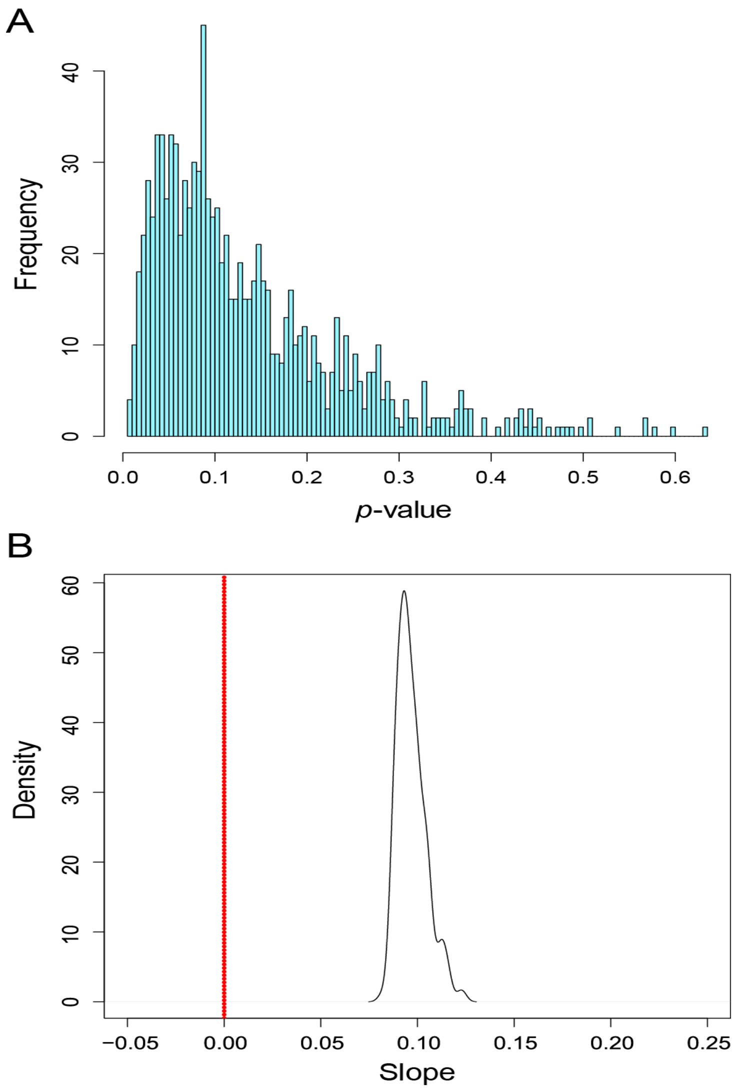

Supplementary Figure S4: Test for the effect of sample selection on model fitting.

Recaptured individuals of known age had more than one sample available for use in regression analyses. In the primary dataset the most recent, and thus oldest, sample from each recaptured was chosen. To ensure no bias was introduced by this selection, 1000 datasets were generated, randomly choosing which sample to use from the recaptured individuals, and then fitting the negative binomial model as before and estimating the significance and size of the age as the sole fixed effect. **A)** shows a histogram of p-values from 1000 models. 19.8% of models predicted a significant association between age and heteroplasmy **B)** shows a density plot of the slopes estimated from the 198 significant models, with a slope of 0 indicated by the red dotted line. The mean estimate was 0.0969 sites per year.

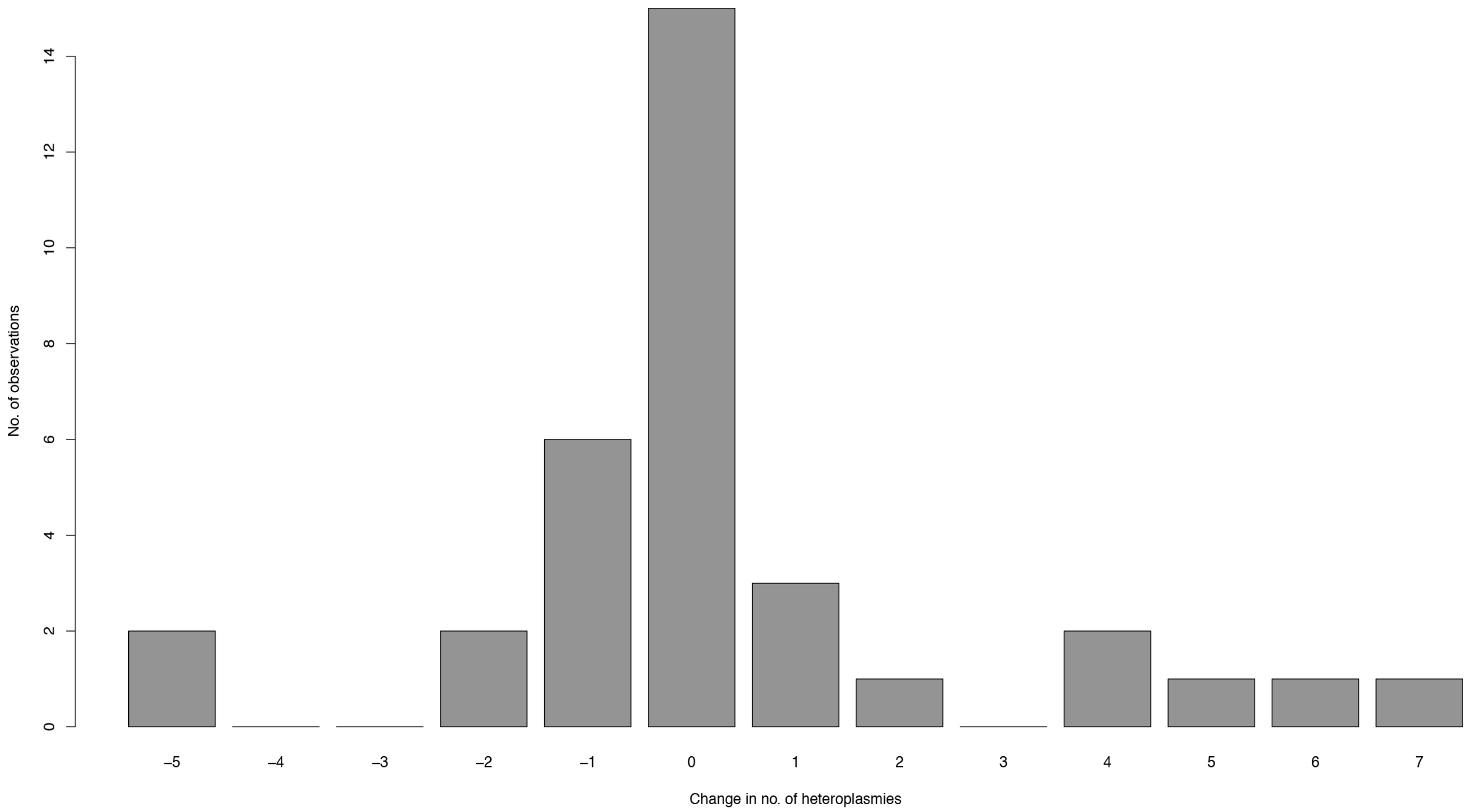

Supplementary Figure S5: Change in the number of heteroplasmy between two consecutive years. Bar plot showing the number of times a change in heteroplasmy was observed, with respect to the size of the change, between two consecutive years. The mean change between any two years was 0.32, with median value of 0, though there were multiple observations of gains/loss of 5 or more heteroplasmies over a year.

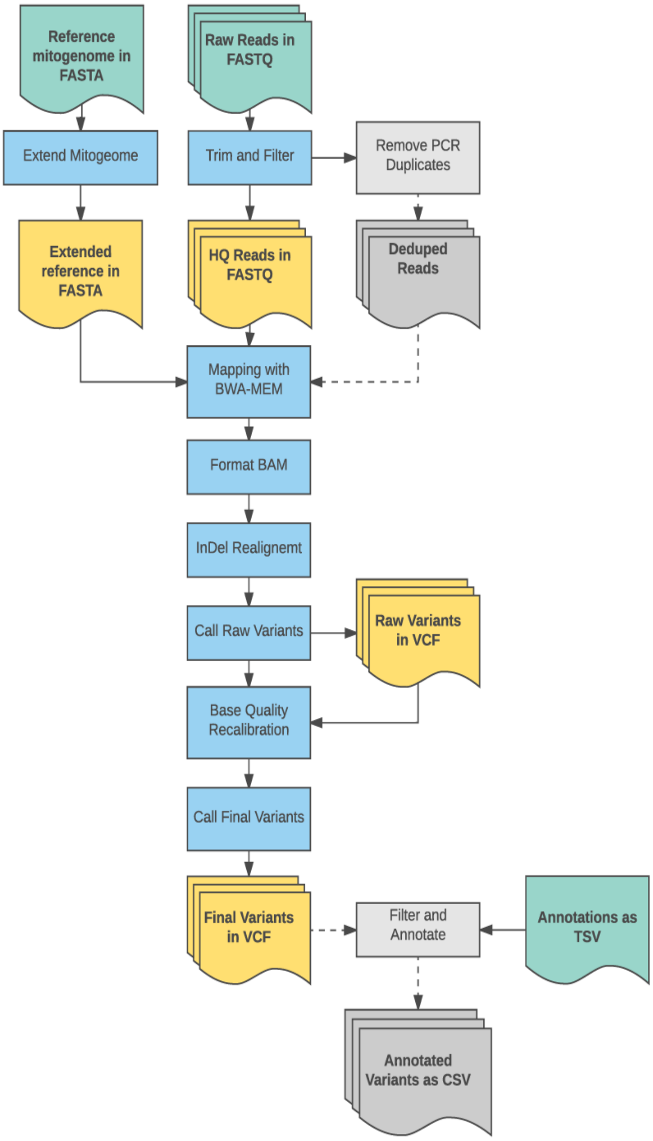

Supplementary Figure S6: Overview of Heteroplasmy Detection Pipeline. Diagram depicting the bioinformatic workflow used in this project. Processes are depicted as rectangles, those in blue are mandatory, while those in grey are optional within the framework of the pipeline (duplicate removal and variant annotation). Documents are represented by rectangles with a wavy base. Those in green are input provided by the user (reference genome, annotations and sequence data), while the yellow documents are generated by default during analyses (extended reference, high quality reads, and variant calls) and grey documents can be generated but are optional (annotated variants). The pipeline can be parallelised, concurrently running a user defined number of jobs.
